# The -Version of the Cramér-von Mises Test for Two-Sample Comparisons in Microarray Data Analysis

**DOI:** 10.1155/BSB/2006/85769

**Published:** 2006-09-14

**Authors:** Yuanhui Xiao, Alexander Gordon, Andrei Yakovlev

**Affiliations:** 1 Department of Biostatistics and Computational Biology, University of Rochester, 601 Elmwood Avenue, P.O. Box 630, Rochester, NY 14642, USA; 2 Department of Mathematics and Statistics, Georgia State University, Atlanta, GA 30303, USA; 3 Department of Mathematics and Statistics, University of North Carolina at Charlotte, 9201 University City Boulevard, Charlotte, NC 28223, USA

## Abstract

Distribution-free statistical tests offer clear advantages in situations where the exact unadjusted -values are required as input for multiple testing procedures. Such situations prevail when testing for differential expression of genes in microarray studies. The Cramér-von Mises two-sample test, based on a certain -distance between two empirical distribution functions, is a distribution-free test that has proven itself as a good choice. A numerical algorithm is available for computing quantiles of the sampling distribution of the Cramér-von Mises test statistic in finite samples. However, the computation is very time- and space-consuming. An  counterpart of the Cramér-von Mises test represents an appealing alternative. In this work, we present an efficient algorithm for computing exact quantiles of the -distance test statistic. The performance and power of the -distance test are compared with those of the Cramér-von Mises and two other classical tests, using both simulated data and a large set of microarray data on childhood leukemia. The -distance test appears to be nearly as powerful as its  counterpart. The lower computational intensity of the -distance test allows computation of exact quantiles of the null distribution for larger sample sizes than is possible for the Cramér-von Mises test.

## 

[1,2,3,4,5,6,7,8,9,10,11,12,13,14,15,16,17,18,19,20,21,22,23,24,25]
